# Retraction Note: Herbal formula GAPT prevents beta amyloid deposition induced Ca^2+^/Calmodulin-dependent protein kinase II and Ca^2+^/Calmodulin-dependent protein phosphatase 2B imbalance in APPV717I mice

**DOI:** 10.1186/s12906-021-03331-0

**Published:** 2021-05-28

**Authors:** Jing Shi, Xuekai Zhang, Long Yin, Mingqing Wei, Jingnian Ni, Ting Li, Pengwen Wang, Jinzhou Tian, Yongyan Wang

**Affiliations:** 1 The 3rd Department of Neurology, Dongzhimen Hospital, Beijing University of Chinese Medicine, Beijing, 100700 China; 2 Key Laboratory of Chinese Internal Medicine, Ministry of Education, Beijing University of Chinese Medicine, Beijing, China; 3 Institute of Clinical Medicine, China Academy of Chinese Medical Sciences, Beijing, China

**Retraction Note: BMC Complement Altern Med 16, 159 (2016)**

**https://doi.org/10.1186/s12906-016-1144-7**

The Editor has retracted this article [1]. Concerns were raised with a number of figures, specifically:

- Figure 1: the panels for Control, APP+D, APP+Gl and APP+Gh appear to be identical to the panels for Control, APP+D, APP+Gl and APP of Fig. 1 [2].
- Figure 2: the bottom right panel appears to be identical with the upper left panel in Fig. 5.
- Figure 4: the upper middle panel appears to be identical with the bottom middle panel of Fig. 5
- Figure 4: the bottom left panel appears to be identical with the bottom left panel of Fig. 5.

The Editor has therefore concluded that the data reported in this article are unreliable.

Jing Shi, Xuekai Zhang, Mingqing Wei, Jingnian Ni, Ting Li, Pengwen Wang and Jinzhou Tian do not agree to this retraction. Long Yin has not responded to any correspondence from the Editor about this retraction. The Editor was not able to obtain a current email address for Yongwan Wang.
